# Light People: Tarik Bourouina

**DOI:** 10.1038/s41377-021-00602-w

**Published:** 2021-08-10

**Authors:** Hui Wang, Xiaoyi Liu

**Affiliations:** 1 Department of International Cooperation, Changchun Institute of Optics, Fine Mechanics and Physics, Chinese Academy of Sciences, 3888 Dong Nan Hu Road, Changchun, 130033 China; 2 ESYCOM Lab, UMR9007 CNRS, Université Gustave Eiffel, 77454 Marne-la-Vallée, France

**Keywords:** Optical materials and structures, Optical physics

## Abstract

In 2015, 195 countries of the United Nations proposed Sustainable Development Goals so as to alleviate the problems of climate change and global pollution. In France, there is a scientist dedicated to contribute providing solutions for above issues by virtue of MEMS, Lab-On-Chip and metamaterials. This expert is Prof. Tarik Bourouina, a Professor of Physics at ESIEE Paris, Université Gustave Eiffel. He devoted himself to the investigations on micro sensors and metamaterials, and kept seeking their applications in the future blueprint of “Sustainable” and “Smart” cities. On the other hand, he has formed an indissoluble bond with *Light: Science and Applications* (LIGHT) since the very beginning of the journal. He also set up the LIGHT’s Paris office, which is the first LIGHT’s overseas office in Europe. We are much honored to have an opportunity to exclusively communicate with Prof. Tarik Bourouina, who will share his research experience and stories with LIGHT in this interview.

## Abstract (web summary)

Prof. Tarik Bourouina devoted himself to the investigations on micro sensors, photonic MEMS and metamaterials, and kept seeking applications in the future blueprint of “Sustainable” and “Smart” cities.

## Graphical abstract

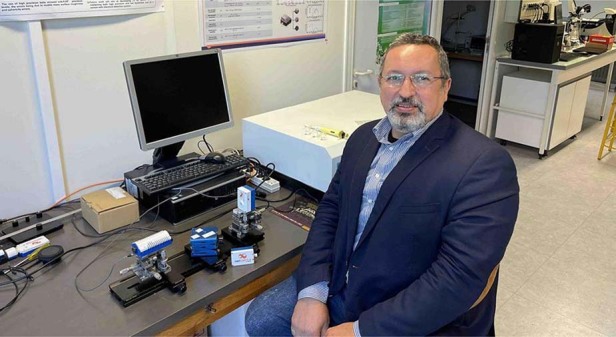



**Biography:** Tarik Bourouina is Professor of Physics at ESIEE Paris, Université Gustave Eiffel. He is affiliated to the French National Center for Scientific Research (CNRS) within the ESYCOM laboratory UMR9007. He was a Senior Research Fellow at The University of Tokyo, Institute of Industrial Sciences, Japan, an associate professor at Université Paris-Saclay, France and Director of a dual-degree Master program in MEMS Engineering with Nanyang Technological University (NTU) in Singapore. His current interests include nano-structured materials, micro-opto-fluidics and their applications to sustainable development. In 2017, he was the recipient of the Chinese Academy of Sciences President’s Fellowship.

Bourouina actively contributed to the launch and development of several companies with his former students and colleagues, which include MEMSCAP, Si-Ware Systems/Neospectra, MEMS-Schlumberger, Fluidion, and Izonics. He has several contributions in Photonic MEMS, among which the smallest MEMS-based FTIR Optical Spectrometer, NEOSPECTRA, developed within the spin-off company Si-Ware-Systems, and was awarded the “Prism Award for Photonics Innovation” in 2014, and the “Technology Showcase Award” in 2017 at the SEMI European MEMS and Sensor Summit.

**1. When did you first learn about the journal** ***Light: Science & Applications*** **(LIGHT)? Why did you agree to be the editor of LIGHT before it became internationally well-known?**

It was in 2012 that I learned from European colleagues that the journal was launched and that there was a very interesting strategy in establishing a top journal dealing with all aspects of light, from the fundamentals to applications. The fact that this journal was co-branded by Nature-Publishing Group and CIOMP, which is among the biggest institutes of Chinese Academy of Science was a very good sign that this journal will be eventually very successful. So, when I was nominated to join the editorial board, I immediately accepted and jumped-in. At that time my main activities were related to optical MEMS and metamaterials including black silicon and other silicon-based metasurfaces. But I was also looking for new research directions. From that perspective, the Light conference was an ideal discussion platform gathering international experts covering a very wide spectrum of optics-related research.

**Figure:**
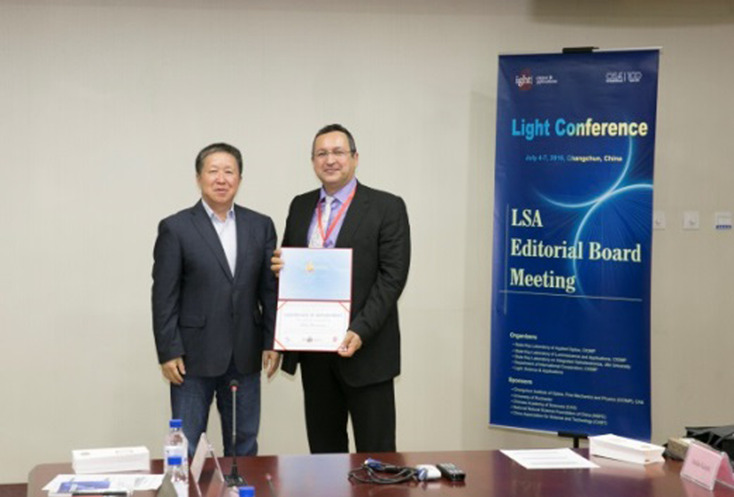
Prof. Jianlin Cao, Editor-in-Chief of journal, awarded the letter of appointment to Professor Tarik in 2016

**Figure:**
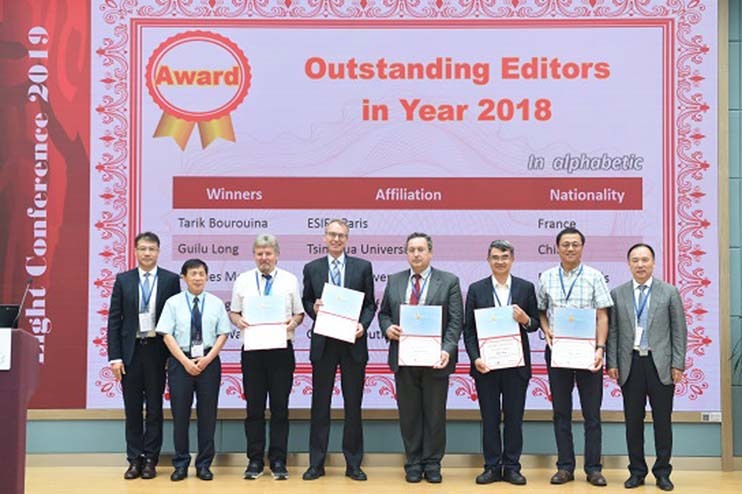
Being awarded the "Outstanding Editors in the Year 2018"

**2. You published a paper on LIGHT in 2013, regarding a novel monolithically integrated silicon micromirror with controlled three-dimensional curvature, which is capable of manipulating optical beams propagating in the plane of the silicon substrate. Could you please give a brief introduction of this work? What are the advantages of the proposed silicon micromirror compared with others?**

Yes this is actually my only paper published in LIGHT to date. The topic deals indeed with the design of 3D-curved mirror whose shape and size can accommodate for multiple applications dealing with free-space propagation of light, especially when considering small spot size and small radii of curvatures of the mirror. One of these applications that we reported later in the same year (in Optics Express) is the ability to produce wide angle spatial optical scanning without deforming the spot size and shape. Our findings were also very useful for the second generation of NEOSPECTRA spectrometer, which operates now with free-space light with no need of optical fiber connectors. This also allowed extending the spectral range up to the Mid-Infrared, which was not possible using optical fibers.

**3. The LIGHT’s overseas office that you set up in your university is its first office in Europe, so could you tell us what kind of work this office is responsible for? What do you think is the significance of setting up LIGHT’s overseas offices all over the world?**

I am proud of being responsible of LIGHT office in Paris. It is indeed hosted by my university. Among the most important events that we had in the recent years is a participation of LIGHT with delegates among the editors and a booth during the international Year in of Light Opening Ceremony hosted in Paris UNESCO headquarters and then during the International Day of Light at UNESCO headquarters in Paris in 2018. Both Prof. Tianhong Cui and Dr. Yuhong Bai attended this event. We had a booth for LIGHT at that occasion and we had the opportunity to meet two Nobel Prizes, Professor Kip Thorne and Professor Claude Cohen-Tannoudji. Besides, we also hosted 12 editors of LIGHT in Paris during the last few years as well as the Vice-President of CIOMP, as part of dissemination actions of LIGHT conducted in France and in Europe.

**Figure:**
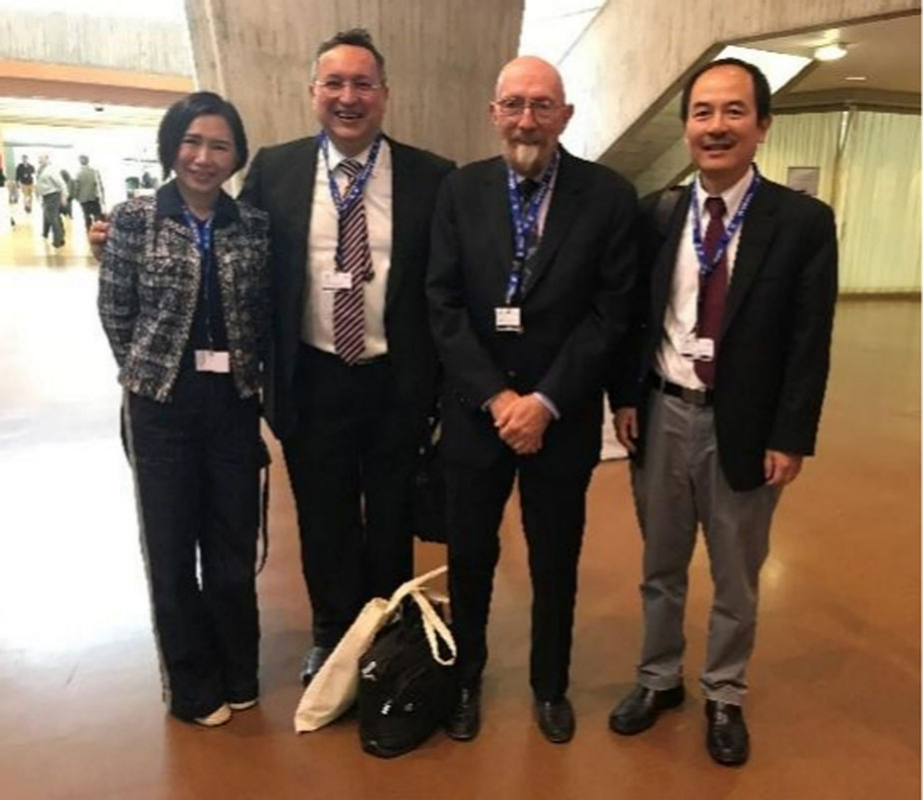
Nobel Prize Kip Thorne with LIGHT Editors Tarik Bourouina, Tianhong Cui and Yuhong Bai during International Day of Light, UNESCO, Paris 2018

**Figure:**
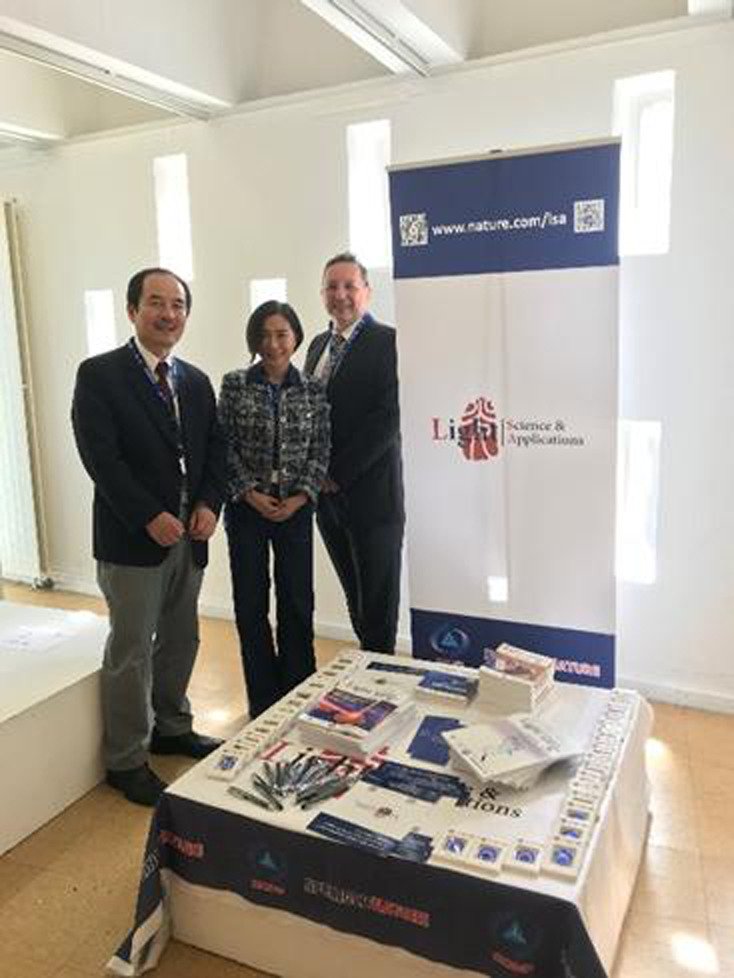
LIGHT Editors Tarik Bourouina and Tianhong Cui and Yuhong Bai near the LIGHT booth at International Day of Light, UNESCO, Paris 2018

**Figure:**
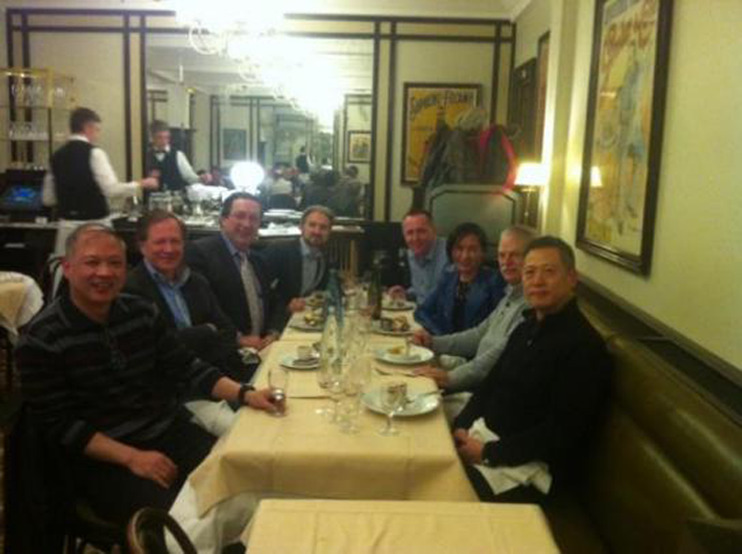
Editors of LIGHT gathering after the opening ceremony of International Year of Light in Paris 2015

**Figure:**
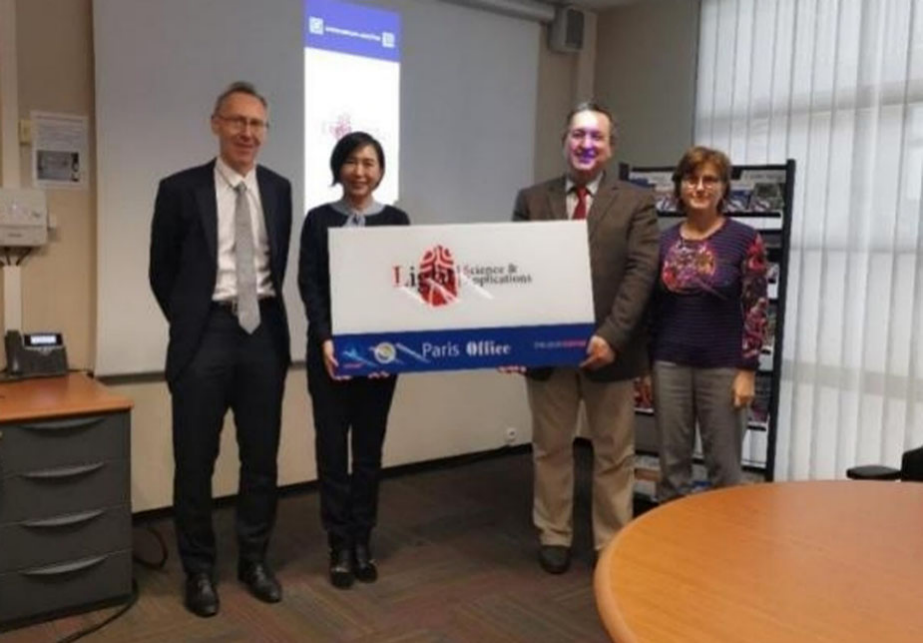
From left to right: Professor Jean Mairesse, Director Yuhong Bai, Professor Tarik Bourouina, and Professor Cécile Delolme

**4. You join the Light Conference almost every year, so would you like to comment this conference?**

Light conference is definitely a key event for sustaining the LIGHT community. That’s why I attended regularly this annual event except last year. I am eager to join the next Light conference in a very next future as soon as possible.

**Figure:**
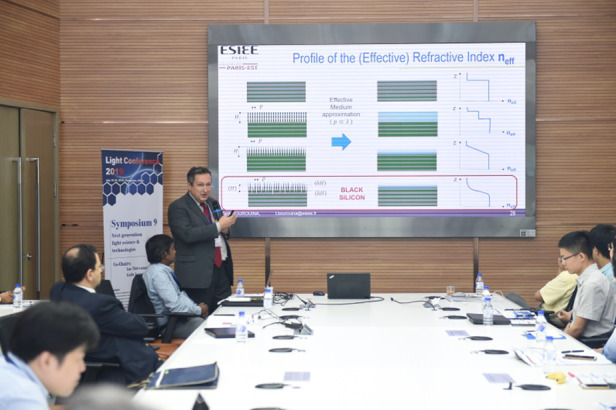
Giving invited talk at Light Conference in 2019

**5. You are an internationally renowned expert in MEMS, Lab-On-Chip and nanomaterials who has obtained a lot of achievements and awards. You won the Prism award on photonics innovation in 2014 by virtue of the smallest MEMS-based FTIR Optical Spectrometer-NEOSPECTRA, jointly developed with Si-Ware-Systems. How did you come up with the idea to develop such a small spectrometer? How does it work? I noticed that this small spectrometer can be further miniaturized, e.g. integrated into our mobile terminals. How will this affect our lives in the future?**

Indeed, besides my academic research activities, I am also engaged since 14 years in Si-Ware Systems, a company established by a former PhD student of my group, Dr. Bassam Saadany. This is very exciting to see the fruit of research efforts turning into a significant impact to the society with this MEMS-based FTIR spectrometer, which is now commercialized under the brand NEOSPECTRA, with tens of thousands units dispatched all over the world. The story started more than 15 years ago with a technology challenge that we tackled using Deep Reactive Ion Etching (DRIE) for the purpose of producing vertical Bragg mirrors, which consists of multiple Silicon-Air layers. In order to satisfy the strict optics requirements, those layers had to be strictly vertical, better than 0,01°, very smooth and deep enough to accommodate with a light spot of 100 microns. After succeeding with this first step, we started exploring the applications. We first made several Fabry-Pérot micro-cavities that were intended to produce tunable filters and refractometers, for instance. Then, during the last year of Bassam’s PhD thesis, we worked on a Michelson interferometer that has a high potential of applications, especially in the FTIR spectrometer architecture. First patent applications were then filed. After that, we have been approached by Hamamatsu Photonics in Japan, considered as the world leading company in photonics and it went very fast to establish a collaboration project that was very beneficial for the young company to develop the first generation of NEOSPECTRA series product line, which is totally owned by Si-Ware Systems. Yes indeed, this product won the prism award in photonics innovation, which was awarded in San Francisco during Photonics West 2014.

**Figure:**
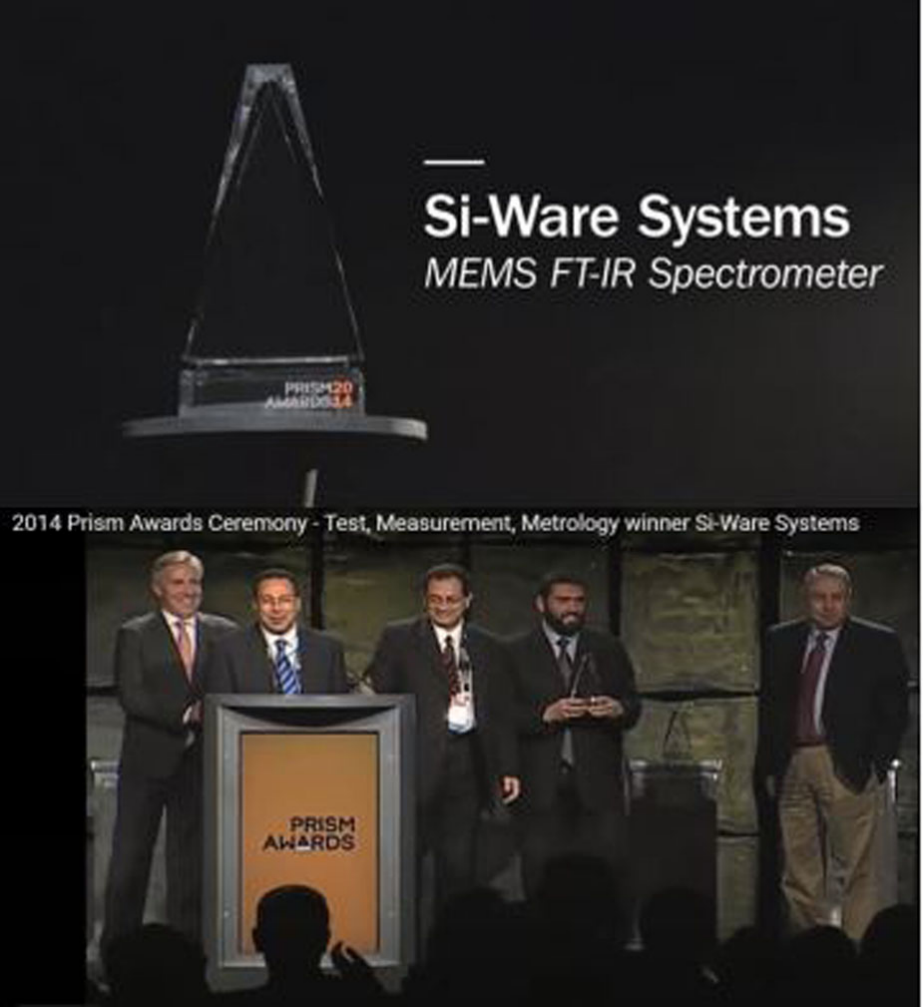

**6. You have been involved in the investigations of Sustainable and Smart Cities based on Lab-On-Chip and micro sensors for several years. What are the definitions of “sustainable” and “smart” here? How can the MEMS and nanomaterials technologies serve in this process? On the other hand, you have always valued the transfer of academic results to industry and market. Could you please tell us your perspective of the potential applications and market prospects of MEMS and nanotechnologies based products?**

Indeed, Sustainable and Smart Cities is my main focus area of applications. There are several reasons for that. One of them is that this is also the flagship focus of my university, so I have to contribute to the collective effort in the frame of the I-Site ‘FUTURE’ Excellence Program supported by the ANR research agency. Second, when you look to the past at the previous success stories of MEMS technologies, you can see that automotive area and smartphones were the main drivers of the huge deployment of MEMS sensors. Based on the current rise of Internet-of-Things (IoT) the next big application areas are definitely Smart Cities and Smart Manufacturing. The corresponding markets are huge and there are a plenty of room for new innovative sensors that do not exist yet. Among these sensors, those dealing with the environment, that is water, air, soil and other natural resources including energy and food. There is a huge avenue for innovation here, with multiple technological challenges and a lot of opportunities for optics technologies before reaching sensors that has to be functionally performant, reliable and low-cost. If one can make them energy-autonomous and biodegradable, this will definitely contribute to both the Smart and Sustainable facets of our efforts. With this, not only MEMS but also Lab-On-Chip and functional nanomaterials can help reaching this contribution to the Sustainable Development Goals, which led to an international Agreement under the United Nations on the Agenda of Sustainable Development for 2030. By the way, this agreement was adopted in Paris in 2015 and it is known as the “Paris Agreement”.

**7. Recently you mainly focused on the studies on nanomaterials and metasurfaces such as Zinc-oxide and black silicon. What kind of remarkable characteristics do they possess? How will they integrate with MEMS and microfluidics systems to develop novel devices which can be utilized in drinkable water generation and purification?**

Yes indeed, Zinc-oxide nanowires are among the nanomaterials that we have interest in. This material has multiple optical properties according to its semiconducting properties, wide-gap and crystalline high quality. For instance it is photochromic and photocatalytic. It is this last property that we have investigated in my group, together with my colleague, Professor Yamin Leprince-Wang. Recently we reported on the integration of ZnO nanowires inside a microfluidic chip for the purpose of water purification. We demonstrated fast removal of Volatile Organic Compounds from water in less than 5 second transit time and also the capability to purify 1 liter of water per day with such tiny chip whose size is one centimeter-square. This work was published in 2018 in Microsystems and Nanoengineering. This work received a best paper award in 2020 from the journal, among the most cited papers within 2 years.

**8. Could you tell us your expectation for LIGHT?**

LIGHT is a wonderful platform, mainly due to the top-level network of experts that are gathering every year during the Light conference. This allows one establishing ties for long-term collaboration. I personally had multiple opportunities to initiate several collaborations after meeting people during the Light conference. This includes collaborations with CIOMP. I had a two-month appointment in 2017 as a laureate of the CAS President Fellowship. I was hosted by Dr. Zhongzhu Liang in the SKAO Lab of CIOMP. More recently, since January 2019, I hosted Dr. Xiaoyi Liu in my lab for a two-year post-doctoral fellowship, which is ending soon. Those two experiences were very enriching and also very fruitful in terms of results.

My expectation from LIGHT is that the “Applications” word which is in the name of the journal could be developed further, not only during the Light conference but also in the journal itself. Indeed, I noticed that recently there were less and less papers being published in LIGHT dealing with Applications. Maybe one way to promote this aspect further is to launch special issues on different themes dealing with applications of LIGHT.

**Comment to the No. 8 Answer**

**Table:** 

Thanks a lot for Prof. Bourouina’s suggestion for LIGHT. In fact, LIGHT has already noticed that when it comes to publishing articles about applications, it’s more difficult than basic or frontier research. In order to solve this problem, LIGHT launched a sister journal in 2020 called *Light: Advanced Manufacturing* (LAM, http://www.light-am.com/), which aims to publish scientific research and application results in the field of advanced manufacturing. LIGHT will try its best to serve the community as always.	

**LIGHT special correspondents**

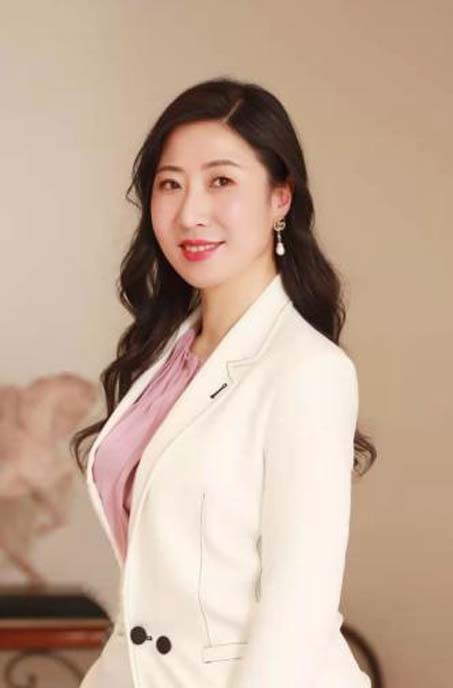



*Hui Wang is the Deputy Director of the Office of International Cooperation in the Changchun Institute of Optics, Fine Mechanics and Physics (CIOMP), Chinese Academy of Sciences (CAS). She currently works on international communication and cooperation for the CIOMP and was a founding member for the Nature Publishing Group and CIOMP joint journal* ***Light: Science & Applications*** *. She is the founder of “Rose in Science” and has published several articles in* ***Acta Editologica, International Talent, Light: Science & Applications*** *, etc., and was invited to take an interview by SPIE Women in Optics, which was published in 2015.*

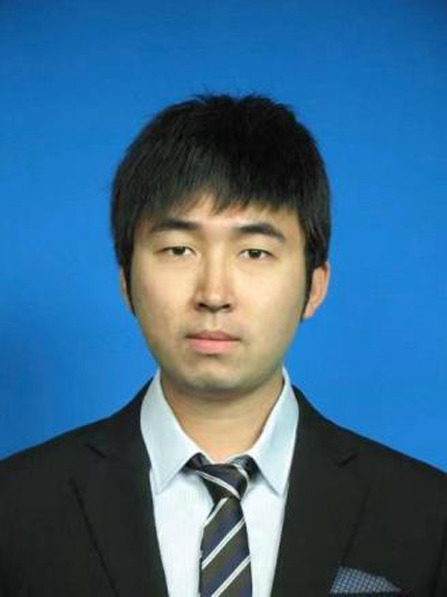



*Xiaoyi Liu is currently a postdoc in ESIEE Paris, ESYCOM Lab, CNRS, Université Gustave Eiffel. He obtained his PhD degree from University of the Chinese Academy of Sciences, Changchun Institute of Optics, Fine Mechanics and Physics in 2018. He joined Prof. Tarik Bourouina’s lab and started his postdoc work from 2019, and was supported by the Chair Blaise Pascal assistant project of École Normale Supérieure Fondation (Supervisor: Prof. Tianhong Cui) and the Future Program of Université Gustave Eiffel in the frame of the I-SITE excellence French national initiative program successively. He once won several honors such as National Scholarship for Postgraduate Students (2015 & 2017), Special Prize of Daheng Optical Scholarship (2017), Special Prize of President Scholarship of Chinese Academy of Sciences (2018), Excellent Graduate Student of Beijing & University of the Chinese Academy of Sciences (2018), Wang Daheng Optical Scholarship for students (2018), Outstanding Doctoral Dissertations of Chinese Academy of Sciences (2019), etc.*

